# *En attendant Godot*: Waiting for the Funeral of “Schizophrenia” and the Baby Shower of the Psychosis Spectrum

**DOI:** 10.3389/fpsyt.2021.618842

**Published:** 2021-05-28

**Authors:** Sinan Guloksuz, Jim van Os

**Affiliations:** ^1^Department of Psychiatry and Neuropsychology, School for Mental Health and Neuroscience, Maastricht University Medical Center, Maastricht, Netherlands; ^2^Department of Psychiatry, Yale University School of Medicine, New Haven, CT, United States; ^3^Department of Psychiatry, Brain Centre Rudolf Magnus, University Medical Centre Utrecht, Utrecht, Netherlands; ^4^Department of Psychosis Studies, King's College London, King's Health Partners, Institute of Psychiatry, London, United Kingdom

**Keywords:** nosology, taxonomy, classification, DSM, early psychosis, schizophrenia

“*Wax on, wax off.”*

From the movie The Karate Kid, 1984

## Introduction

The debate on the concept of schizophrenia is alive and kicking (1) because the concept of schizophrenia is dead and decaying (2). Despite continual demands for reconceptualization proposed by highly influential academics, heated discussions during the revision processes of DSM and ICD, and attacks from every angle, the concept of schizophrenia, as we know it, has managed to “make a goal-line stand” every time. These discussions over decades have failed to go beyond merely stimulating exchanges between scholars that resulted in minor revisions only. We—like the two characters in *En attendant Godot*—are still waiting for a meaningful action toward reconceptualization that probably will never come. In this brief viewpoint, we will attempt to summarize the shortcomings of the schizophrenia concept and reiterate our understanding of psychosis spectrum disorder—hopefully, once and for all.

## The Illusion of Etiological Specificity

The evidence thus far suggests that the etiology of mental disorders consists of multicausal, interdependent, interacting, and non-specific factors contributing to largely shared behavioral, social, and biological mechanisms (3). Schizophrenia is no exception.

Environmental factors, as part of a dynamic network (so-called exposome), associated with schizophrenia, are interdependent and causally and non-causally related to almost all psychiatric phenotypes (4). In the general population, environmental exposures, such as cannabis use and childhood adversity, are not only directly associated with psychotic experiences but also interact with multidimensional psychopathology and family history of affective disorders to increase psychosis expression: the so-called affective pathway to psychosis (5–7).

Genome-wide association studies (GWAS) consistently demonstrate that schizophrenia is genetically correlated with various psychiatric disorders, in particular bipolar disorder (8, 9). Similarly, polygenic liability score for schizophrenia is non-specifically associated with subclinical multidimensional phenotypes, including cognitive and affective domains (10–13), as well as broad mental and physical health outcomes in the general population (14).

Based on the findings showing phenomenological, cognitive, genetic, molecular, and electrophysiological similarities between schizophrenia and bipolar disorder (15), we have argued that schizophrenia, schizoaffective disorder, and bipolar disorder may be different phenotypic presentations of a largely shared pathoetiology with diverse outcome trajectories (2). Multiple sclerosis is a well-known example of substantial phenotypic and clinical heterogeneity that stems from a shared pathoetiology. With the analogy of multiple sclerosis, these different diagnostic categories might be different types of a shared disease process with varying outcomes and phenotypical representations, suggestive of a unitary model of psychosis instead of discrete entities such that: brief psychotic disorder ~ clinically isolated syndrome; bipolar disorder ~ relapsing-remitting type; schizoaffective disorder ~ secondary progressive type; schizophrenia ~ primary progressive type. In light of accumulating evidence, we contemplate that psychosis spectrum disorder, a superordinate level category, would likely encompass bipolar disorder, at least in clinical research practice that we already observe in contemporary first episode psychosis studies. However, more transdiagnostic research is needed to confirm this proposition. Furthermore, we wish to clarify that this unitary framework should not be interpreted as against the possibility of distinct subtypes. We envision this unitary approach will set the ideal stage to think beyond the borders of traditional categories in the pursuit of improved taxonomy and may eventually lead to more precise classification.

## The Illusion of Discrete Entity

The current taxonomy implies that schizophrenia represents a point of rarity, a discrete disease phenotype with well-defined boundaries. However, converging evidence suggests that psychosis expression, including positive, negative, and cognitive symptoms, represents an etiologically, phenomenologically, and temporally continuous phenotype across the general population, with prevalence rates varying between 5% (interview-rated) and 8% (self-report) (16, 17).

In the temporal domain, subclinical psychosis expression is associated with subsequent clinical psychotic disorders and non-psychotic disorders (18) and functional impairment, serving as a general severity indicator for broad psychopathology (17).

Recent findings from GWAS provide support to the liability-threshold model, first postulated by Gottesman and Shields more than 50 years ago (19). According to this model, which is fully compatible with the psychosis continuum concept, each individual has quantifiable (environmental and genetic) liability for schizophrenia to varying degrees but develops schizophrenia only when the combined liability exceeds the threshold on the continuum. Conforming to the psychosis continuum model, polygenic risk score for schizophrenia is associated with psychotic experiences in the general population (13). Furthermore, recent evidence lends support for a shared genetic liability between schizophrenia and psychotic experience (20). Environmental factors associated with schizophrenia—childhood trauma, cannabis use, urban environment—are likewise strongly associated with psychotic experiences at the population level (21). Furthermore, recent studies suggest that genetic liability for schizophrenia interact with environmental exposure to increase psychosis expression and comorbid psychopathology (22, 23).

## The Illusion of Phenotypic Specificity

Per definition of current classifications, schizophrenia represents a true distinct disease entity, of which the boundaries are clearly defined. This implication of rarity has reassured the implicit confidence of “schizo”-prism that the origins of the prodrome can logically be traced back using the same operational criteria, with a particular emphasis on positive psychotic subclinical symptoms. This unfounded confidence has led to the birth of the “clinical high risk” concept (24). However, it appears that the predictive performance of the clinical high risk is low, with only around 15% transitioning to clinical psychosis over a 3-year period in the help-seeking population (25). The fixation on psychosis—disregarding early expression of non-specific symptoms—comes at the expense of the multidimensional nature of psychopathology. However, it is well-established that non-psychotic psychopathology, such as anxiety, depressed mood, sleep disturbance, motivational impairment, social and neurocognitive alterations, precede early stages of psychotic disorders—so called heterotypic continuity.

In fact, the population-based estimates clearly show that even though the psychosis high-risk state displays a high relative risk for subsequent clinical psychosis outcome, the incidence of clinical psychosis outcome in the general population is largely attributable to non-psychotic mental disorder categories (i.e., mood, anxiety, alcohol, and drug use disorders) (18). These findings show that targeted “clinical high risk” early intervention model based on the schizophrenia concept can yield minimal benefit at the expense of major resource for case-finding, considering the scarcity of the psychosis high-risk state in the population (24, 26).

## The Illusion of Poor Outcome

Per definition, schizophrenia is associated with chronicity, deterioration, and poor outcome—as reflected by psychiatrists' perception of schizophrenia: “Persons that turn out ‘normal’ again a few years later, I am forced to consider that I was mistaken about a schizophrenia early diagnosis” (27); “Good prognosis ‘schizophrenia’ is not mild schizophrenia, but a different illness” (28). In fact, studies show that a major challenge for improving the outcome of schizophrenia is paradoxically the narrow definition of neo-Kraepelinian schizophrenia, first introduced in DSM-III (29, 30).

Furthermore, accumulating evidence shows that early studies conducted mainly in inpatient units and tertiary specialized centers typically collect severity- and chronicity-enriched samples of patients with poor outcome and therefore are subject to systematic selection bias that is known as Berkson's bias (31). In this regard, early studies of enriched samples overlook patients with better outcome and those recovered or displayed an improved course of illness and thereby no longer meeting the criteria for schizophrenia diagnosis. Findings from the contemporary studies, particularly those from the follow-up of patients with first episode psychosis in early intervention services, demonstrate that better outcomes are achievable (32). The 10-year follow-up of the Scandinavian TIPS Early Detection in Psychosis Study demonstrated that the recovery percentage was significantly higher in early-detection patients than those in the usual-detection area (30.7 vs. 15.1%) (33).

## The Illusion of Clinical Utility

Psychiatry has disproportionately and erroneously placed too much emphasis on the clinical utility of diagnoses (34). As discussed above, schizophrenia diagnosis does not provide testable theories about the pathoetiology, treatment planning, or management but only “moves the goalpost” with the claim of predicting the course, which in reality comes with the ingrained chronicity and deterioration into the definition of schizophrenia.

Schizophrenia diagnosis has largely been deemed fairly stable and definitive, but mental health care professionals report that inaccurate and controversial diagnosis of schizophrenia in their clinical practice takes place frequently (35). Accordingly, the results of a WHO survey demonstrate that clinicians rate the ease of use and goodness of fit of schizophrenia no higher than other diagnoses, such as depressive and bipolar disorders (36).

## Solutions for Illusions

There is a growing dissatisfaction with the notion of reifying psychiatric diagnostic categories as discrete entities. Research in search of the origins of schizophrenia has yielded neither actionable nor tangible evidence to improve our understanding. Several frameworks alternative to categorical conceptualization have been introduced particularly for research purposes: the US National Institute of Mental Health (NIMH) initiated Research Domain Criteria (RDoC) and Hierarchical Taxonomy of Psychopathology (HiTOP). Although the multidimensional assessment of schizophrenia was introduced in Section III of DSM−5 as “emerging measures” and the wording was revised slightly as “schizophrenia spectrum disorder,” these changes had minimal impact on our use of schizophrenia in clinical practice.

It is clear that we need more—much more—evidence to propose drastic changes in the nosology of mental disorders including schizophrenia. Therefore, instead of a “grand idea,” we propose a modest solution to pave the way for better conceptualization and improving clinical practice by emphasizing the importance of clinical characterization over diagnostic reductionism (37, 38). To encourage clinicians and researchers to think outside the borders of schizophrenia, we embrace a trans-syndromal framework of mental suffering yet retain an “umbrella” syndrome category (psychosis spectrum disorder) to satisfy clinical practice conventions (2). In fact, we propose the following framework: *psychosis spectrum* + *clinical characterization* (38). The use of “psychosis spectrum,” while nomothetic, deliberately refers to something so broad and non-specific that it only makes sense if it is accompanied by an idiographic personal characterization. As the word “schizophrenia” has indelible negative connotations and implicit support for discrete entity, renaming is essential to enable seeing without the imaginary boundaries of current schizophrenia concept (39).

## Meta-Solutions for Denial: From Repudiation to Taking Responsibility

It is clear that the time for the funeral of schizophrenia was yesterday; nevertheless, we remain in the denial stage. Why is this so?

About three decades ago, Mary Boyle wrote her seminal work on schizophrenia as a “scientific delusion” (40). Many authors have since delivered similar cogent, scientific, clinical, ethical, and public health arguments for abandoning the schizophrenia concept (41–45)—yet nothing has changed. It is well-known that a switch in terminology can result in a disease being perceived as more serious and more likely to be a rare condition (46). Therefore, in medicine, changes in terminology are readily applied in response to social or ethical demands. Erectile Dysfunction, Myocardial Infarction, Alzheimer's disease and Down's syndrome are but a few examples. Such changes reflect the advent of the “moral era” of medicine and health care (47), in which the focus is not on narrow medical outcomes *per se* but on the degree to which they add value to highly personal life goals of the patient. Patients, professionals, and institutions therefore should learn to work together to “co-create” a terminology to suit the needs of the individual and society within the space of the inevitable scientific uncertainty surrounding the condition in question. Arguably, no area of medicine presents with more moral dilemmas as the practice of calling mental variation “things”—for example, “schizophrenia”—particularly, if accompanied by scientifically unfounded conviction that the “thing” is a nosological entity and is embedded exclusively in the brain. The bearer of an experience that falls within this nosological entity, for example, a person hearing voices, likely will have difficulties making himself “heard,” because the mental health professional—and society in line with him—hears a symptom of a distinct brain disease. This phenomenon is called “epistemic injustice” and arguably represents one of the most important dilemmas to solve, should psychiatry wish to enter the moral era of medicine (48). Put simply, pre-mature conclusions based on inconclusive science have real consequences that can result in epistemic injustice, and the use of the term “schizophrenia” has all the hallmarks of this. The degree to which psychiatry remains tone deaf to the issue of epistemic injustice inherent to schizo-labeling, matches with the evident loss of societal support for psychiatry as a science (49). Psychiatry, unlike oncology for example, receives cogent and well-organized critical feedback from many sources, including *Mad in America* and the *Hearing Voices Movement*. Instead of ignoring these sources of critical review, psychiatry could actively engage with them and co-create solutions, particularly for pressing problems like the language and the concepts we use to describe mental variation.

In conclusion, there is ample reason for psychiatry to consider the issue of management of diversity with the gravity it deserves. Instead of letting the field become increasingly imprudent to diversity, we can choose innovation that befits the moral era of medicine, and grow out of our self-imposed state of non-responsiveness to embrace diversity in a fashion that fits science and avoids epistemic injustice.

## Author Contributions

SG and JO contributed to the article and approved the submitted version.

## Conflict of Interest

The authors declare that the research was conducted in the absence of any commercial or financial relationships that could be construed as a potential conflict of interest.
